# Nutritional status and body composition in cognitively impaired older persons living alone: The Takashimadaira study

**DOI:** 10.1371/journal.pone.0260412

**Published:** 2021-11-23

**Authors:** Masanori Iwasaki, Keiko Motokawa, Yutaka Watanabe, Misato Hayakawa, Yurie Mikami, Maki Shirobe, Hiroki Inagaki, Ayako Edahiro, Yuki Ohara, Hirohiko Hirano, Shoji Shinkai, Shuichi Awata

**Affiliations:** 1 Tokyo Metropolitan Institute of Gerontology, Tokyo, Japan; 2 Faculty of Dental Medicine, Gerodontology, Department of Oral Health Science, Hokkaido University, Sapporo, Japan; 3 Kagawa Nutrition University, Saitama, Japan; Ehime University Graduate School of Medicine, JAPAN

## Abstract

**Objectives:**

To investigate nutritional status and body composition in cognitively impaired older persons living alone.

**Methods:**

This cross-sectional study included 1051 older adults (633 women and 418 men, mean age: 77.1 years) from the Takashimadaira study. The study participants were categorized according to whether they lived alone, which was confirmed via questionnaire, and had cognitive impairment, which was defined as having a Mini Mental State Examination-Japanese score ≤23. Nutritional status was evaluated using the serum albumin level. The fat-free mass index (FFMI) was calculated based on anthropometric and body composition measurements. A logistic regression model with the outcome of a low serum albumin level (serum albumin <4 g/dL) and low FFMI (<16 kg/m^2^ in men and <14 kg/m^2^ in women) were used to analyze the data.

**Results:**

The percentages of participants in the living alone (-)/cognitive impairment (-) group, the living alone (+)/cognitive impairment (-) group, the living alone (-)/cognitive impairment (+) group, and the living alone (+)/cognitive impairment (+) group were 54.8%, 37.3%, 5.6%, and 2.3%, respectively. Compared to the living alone (-)/cognitive impairment (-) group, the living alone (+)/cognitive impairment (+) group was more likely to have a low serum albumin level (adjusted odds ratio = 3.10, 95% confidence interval = 1.31 to 7.33) and low FFMI (adjusted odds ratio = 2.79, 95% confidence interval = 1.10 to 7.06) after adjusting for potential confounders.

**Conclusion:**

Cognitively impaired older adults living alone had poorer nutrition than cognitively normal and cohabitating persons in this study. Our results highlight the importance of paying extra attention to nutritional status for this group of community-dwelling older adults.

## Introduction

Malnutrition can result from a lack of intake or uptake of nutrition, disease, or inflammation, alone or in combination [1]. Malnutrition is common in older adults. It leads to altered body composition (decreased fat free mass) [1] and increases the risks of frailty, sarcopenia, morbidity, and mortality [2–6].

Advancing age is strongly related to declines in cognitive function [7]. There are increasing numbers of older adults with cognitive impairment in Japan, which has the world’s highest life expectancy alongside an increasingly aging population [8]. In terms of their living arrangements, the number of people with cognitive impairment who live alone is not low [9, 10]. According to a survey on the status of households including cognitively impaired persons in Tokyo, Japan, 1 in every 6 cognitively impaired adults was found to live alone [9].

Both cognitive impairment and living alone have been reported to be associated with poor nutritional status [11–15]. To date, many analyses of malnutrition associated with cognitive impairment and living alone have been performed, but they have examined cognitive impairment and living alone separately [11–15]. Therefore, the nutritional status of cognitively impaired older persons living alone is unknown. If the nutritional status of cognitively impaired older persons living alone is identified as poor, it might be possible to pay extra attention to their nutritional care. In addition, such insights may in turn lead to new strategies to support nutritionally vulnerable older adults who wish to remain in their own homes as long as possible, even if they live alone with cognitive impairment.

In this cross-sectional study, we aimed to investigate nutritional status and body composition in cognitively impaired older persons living alone. We hypothesized that cognitively impaired older persons living alone would be more likely to have poor nutritional status and decreased fat-free mass than cognitively normal and cohabitating persons.

## Materials and methods

### Study design, setting, and population

This cross-sectional study was based on the Takashimadaira study [16], a community-based study that was conducted in 2016, with the aim of developing a model for dementia-friendly communities in a metropolitan area. First, a questionnaire on living arrangements, demographic and socioeconomic status, health behavior, and health status was mailed to individuals aged ≥70 listed in the basic resident register of Takashimadaira, Itabashi Ward, Tokyo, Japan. Thereafter, the investigators of the Takashimadaira study obtained the completed questionnaires through home visits. During the home visits, the investigators asked the targeted adults about their willingness to participate in a comprehensive health assessment to be conducted at a healthcare facility. Those who agreed underwent a comprehensive health assessment, which included a cognitive assessment, nutritional assessment, anthropometric and body composition measurement, medical interview, and oral examination. For the current investigation, the Takashimadaira study participants with incomplete data were excluded from the analysis.

The Takashimadaira study was conducted in accordance with the ethical principles of the Declaration of Helsinki and was approved by the Ethics Committee of the Tokyo Metropolitan Institute of Gerontology (Approval number: 9 and 31 in 2016). Upon enrollment, all individuals were given a detailed explanation about the purpose of the research, voluntary participation, the study protocol, the potential risks and benefits, confidentiality, and the right to refuse or withdraw. We considered all the individuals who visited a healthcare facility to receive a comprehensive health assessment and who were informed of the study, had the capacity to provide consent. The Ethics Committee of the Tokyo Metropolitan Institute of Gerontology approved this procedure for recruiting the study participants. After the explanation, the individuals provided written informed consent prior to participation in the study. All data/samples were fully anonymized before we accessed them. Because this study was designed as a secondary analysis of the Takashimadaira study, no a priori sample size calculation was performed.

### Questionnaire survey

Data on participants’ living arrangements, age, sex, educational status (i.e., years of schooling), annual income, smoking status, alcohol consumption, physical activity level, appetite, social isolation, instrumental activities of daily living (IADL), and depressive symptoms were obtained through a self-administered questionnaire that was mailed before the health examinations. The detailed definitions for each variable of the questionnaire survey are summarized in Table 1.

**Table 1:** Variables measured in the questionnaire survey and medical interview.

Variable	Type of variable	Definition
Living arrangements	2-level categorical	Living alone or not
Age	Continuous	Age in years
Sex	2-level categorical	Man or woman
Educational status	Continuous	Years of schooling
Annual income	2-level categorical	Annual income of less than 3 million Japanese Yen or not [17]
Smoking status	2-level categorical	Current smoker or not
Alcohol consumption	2-level categorical	Daily drinker or not
Physical activity levels	2-level categorical	Low level (do not engage in physical exercise or sports) or not low level
Appetite	2-level categorical	Poor appetite (CNAQ score ≤ 28) or not [18]
Social isolation	2-level categorical	Yes (participants answered “no” to the question “Do you have contact at least once a week with anyone including relatives living apart, friends, and neighbors?”) or no [19]
IADL	Continuous	Assessed using the JST-IC, with higher scores indicating greater competence [20]
Depressive symptoms	2-level categorical	Yes (GDS-15 score ≥5) or no [21, 22]
Comorbidity status	2-level categorical	Present or absent for each of four comorbidities (heart disease, stroke, diabetes, and cancer)
History of hospitalizations within the prior 12 months	2-level categorical	Yes or no
Certification for LTCI	2-level categorical	Obtained a certification for LTCI or not

CANQ = Council on Nutrition Appetite Questionnaire, GDS-15 = the Japanese version of the 15-item Geriatric Depression Scale, IADL = instrumental activities of daily living, JST-IC = the Japan Science and Technology Agency Index of Competence, LTCI = long-term care insurance.

### Cognitive assessment

The participants underwent the Mini Mental State Examination-Japanese (MMSE-J) [23], which was administered by trained nurses and clinical psychologists. Study participants with MMSE-J scores ≤23 were defined as having cognitive impairment.

### Serum albumin measurement

Serum albumin levels were measured as an indicator of nutritional state by a modified bromocresol purple method using an automatic biochemical analyzer (JCA-BM8060; JEOL, Ltd., Tokyo, Japan). A serum albumin level ≤4.0 g/dL was defined as low [24, 25].

### Anthropometric and body composition measurement

Weight and height were measured with the participant wearing light clothing but no shoes. The body mass index was computed by dividing the weight in kilograms by the squared height in meters (kg/m^2^). Fat-free mass, fat mass, 50 kHz phase angle, total body water (TBW), and extracellular water (ECW) were measured using the body composition analyzer InBody S10 (Biospace, Seoul, Korea) with the study participants in a seated position. Absolute fat-free mass was converted to the fat-free mass index (FFMI) by dividing it by the squared height in meters (kg/m^2^). FFMI <16 kg/m^2^ in men and <14kg/m^2^ in women were defined as low [26]. Furthermore, the fat mass index [FMI (kg/m^2^); absolute fat mass divided by the squared height in meters], TBW per unit of weight, and ECW per unit of TBW were calculated.

### Medical interviews

Well-trained study staff confirmed the participants’ comorbidity status, history of hospitalizations within the prior 12 months, and status of long-term care insurance (LTCI) certification. Four comorbidities were identified, which included heart disease, stroke, diabetes, and cancer. Regarding the certification of LTCI, the study participants were categorized into one of following categories; no certification, requiring support levels 1 or 2, or requiring long-term care level 1, 2, 3, 4, or 5. In our study population, 3.7% of the participants were certified as requiring support level 1, 1.8% required support level 2, 1.1% required long-term care level 1, 0.3% required long-term care level 2, and no one required long-term care of level 3 or higher. Because only 6.9% obtained an LTCI certification, they were combined into one category (Table 1).

### Oral examination

Chewing and swallowing abilities were assessed by trained dentists and dental hygienists. Chewing ability was assessed using gummy jelly (test gummy jelly for evaluating chewing performance; UHA Mikakuto Co., Ltd., Osaka, Japan). The participant was asked to chew a gummy jelly for 30 strokes. Dentures, when used, were left in for assessment. The degree of fracture in the chewed gummy jelly was evaluated through comparison with the visual reference material and was assigned a score ranging from 0 to 9, where a higher score indicated better chewing ability. Study participants with gummy jelly scores ≤2 were defined as having low chewing ability [27].

Swallowing ability was assessed using the repetitive saliva swallowing test (RSST) [28]. Each participant was asked to swallow saliva as many times as possible for 30 seconds, while the number of swallows was counted through palpation of the larynx. Study participants with swallow counts of ≤2 were defined as having low swallowing ability.

### Statistical analyses

The primary outcomes included a low serum albumin level (dichotomous variable: presence or absence) and low FFMI (dichotomous variable: presence or absence). Additionally, having both a low serum albumin level and low FFMI was set as another outcome. The principal exposure variables were living arrangements and cognitive status. Based on these two exposure variables, study participants were divided into four groups: participants who were not living alone or cognitively impaired (living alone (-)/cognitive impairment (-)); participants who were living alone but were not cognitively impaired (living alone (+)/cognitive impairment (-)); participants who were not living alone but were cognitively impaired (living alone (-)/cognitive impairment (+)); and participants who were both living alone and cognitively impaired (living alone (+)/cognitive impairment (+)).

The study population characteristics according to living arrangements and cognitive status were described using analysis of variance, the Kruskal–Wallis test, and the chi-squared test as appropriate. If the overall test was significant, post hoc comparisons were performed. The Shapiro–Wilk test was used to determine whether the continuous variables were normally distributed.

The crude and multivariable adjusted odds ratios (ORs) of a low serum albumin level and low FFMI according to living arrangements and cognitive status were calculated through logistic regression analyses. Based on previous studies [29–34], the following variables were considered covariates: age, sex, educational status, annual income, smoking status, alcohol consumption, physical activity level, chewing and swallowing abilities, appetite, social isolation, IADL, depressive symptoms, number of comorbidities, the history of hospitalizations, and the status of LTCI certification.

Furthermore, instead of using a 4-categorical variable based on the presence of living alone and cognitive impairment, the joint effects between living alone and cognitive impairment were evaluated by adding interaction terms in the regression models.

Analyses were performed with the statistical software package STATA version 17.0 (StataCorp, College Station, TX, USA). The level of significance (two-tailed test) was set to 0.05.

## Results

In 2016, a survey questionnaire was sent to 7,614 residents aged ≥70. Of these, 5,430 (71.3%) returned their completed questionnaire. They were additionally invited to participate in the comprehensive health assessment, wherein 1,248 individuals (23.0%) responded positively and underwent the assessment. Among them, 197 did not submit complete data and were thus excluded. The remaining 1051 adults [633 women (60.2%) and 418 men (39.8%)] were included in the current analysis. The age range was 70 to 96 years, with a mean [standard deviation (SD)] of 77.1 (4.7) years.

There were 576 adults (54.8% of the study population) belonging to the living alone (-)/cognitive impairment (-) group, 392 (37.3%) to the living alone (+)/cognitive impairment (-) group, 59 (5.6%) to the living alone (-)/cognitive impairment (+) group, and 24 (2.3%) to the living alone (+)/cognitive impairment (+) group. Table 2 presents the comparison of study participant characteristics according to living arrangements and cognitive status. The copresence of living alone and cognitive impairment was associated with a low MMSE-J score, low serum albumin level, shorter height, low TBW, low ECW, low FFMI, advanced age, low education, low chewing ability, low swallowing ability, low Japan Science and Technology Agency Index of Competence score, and high frequency of copresence of a low albumin level and low FMI and LTCI certification.

**Table 2:** Characteristics of the study population according to living arrangements and cognitive status.

	Total	Living alone (-) Cognitive impairment (-)	Living alone (+) Cognitive impairment (-)	Living alone (-) Cognitive impairment (+)	Living alone (+) Cognitive impairment (+)	
	N = 1,051	N = 576	N = 392	N = 59	N = 24	
	100%	54.8%	37.3%	5.6%	2.3%	p-value
MMSE-J*	28 (26–29)	28 (27–29)^a^	28 (27–29)^a^	22 (20–23)^b^	22 (19–23)^b^	<0.01
Serum albumin level (g/dl)^†^	4.2 (0.3)	4.2 (0.3)	4.2 (0.3)	4.1 (0.3)	4.1 (0.3)	0.05
Low serum albumin level^‡^	298 (28.4%)	144 (25.0%)	120 (30.6%)	22 (37.3%)	12 (50.0%)	0.01
Anthropometric and body composition						
Height (cm)^†^	155.6 (8.6)	157.4 (8.7)^a^	153.1 (7.6)^b^	155.5 (9.4)^a^^,^ ^b^	151.4 (8.6)^b^	<0.01
Weight (kg)^†^	55.9 (10.2)	57.4 (10.2)^a^	53.9 (9.7)^b^	56.3 (11.7)^a^^,^ ^b^	52.5 (8.5)^a^^,^ ^b^	<0.01
BMI (kg/m^2^)^†^	23.0 (3.2)	23.1 (3.2)	22.9 (3.3)	23.1 (3.6)	22.8 (2.6)	0.85
FMI (kg/m^2^)^†^	7.0 (2.4)	6.7 (2.4)^a^	7.3 (2.4)^b^	7.3 (2.7)^a^^,^ ^b^	7.4 (2.3)^a^^,^ ^b^	<0.01
Phase angle (°)^†^	4.7 (0.7)	4.8 (0.6)^a^	4.6 (0.6)^b^	4.6 (0.7)^b^	4.5 (0.6)^a^^,^ ^b^	<0.01
TBW (l)^†^	28.8 (5.5)	30.0 (5.6)^a^	27.1 (4.8)^b^	28.6 (5.9)^a^^,^ ^b^	26.1 (4.7)^a^^,^ ^b^	<0.01
TBW/wt (%)^†^	51.7 (5.2)	52.5 (5.1)^a^	50.6 (5.0)^b^	51.0 (5.6)^a^^,^ ^b^	50.0 (5.2)^a^^,^ ^b^	<0.01
ECW (l)^†^	11.2 (2.1)	11.6 (2.1)^a^	10.5 (1.8)^b^	11.1 (2.2)^a^^,^ ^b^	10.2 (1.8)^a^^,^ ^b^	<0.01
ECW/TBW (%)^†^	38.8 (0.7)	38.7 (0.7)^a^	38.9 (0.7)^b^	39.1 (0.8)^b^	39.1 (0.7)^a^^,^ ^b^	<0.01
FFMI (kg/m^2^)^†^	16.1 (1.7)	16.4 (1.7)^a^	15.7 (1.6)^b^	15.9 (1.8)^a^	15.4 (1.3)^a^^,^ ^b^	<0.01
Low FFMI^‡^	168 (16.0%)	77 (13.4%)	67 (17.1%)	16 (27.1%)	8 (33.3%)	<0.01
Low serum albumin level and low FFMI^‡^	61 (5.8%)	22 (3.8%)	25 (6.4%)	8 (13.6%)	6 (25.0%)	<0.01
Others						
Age^†^	77.1 (4.7)	76.4 (4.4)^a^	77.7 (4.8)^b^	78.7 (5.8)^b^	79.1 (4.9)^a^^,^ ^b^	<0.01
Sex^‡^						<0.01
Women	633 (60.2%)	290 (50.3%)	298 (76.0%)	29 (49.2%)	16 (66.7%)	
Men	418 (39.8%)	286 (49.7%)	94 (24.0%)	30 (50.8%)	8 (33.3%)	
Educational status (years of schooling)*	12 (12–15)	12 (12–16)^a^	12 (11–14)^b^	12 (9–16)^bc^	12 (9–12)^c^	<0.01
Annual income < 3 million JPY^‡^	667 (63.5%)	293 (50.9%)	329 (83.9%)	28 (47.5%)	17 (70.8%)	<0.01
Current smoker^‡^	68 (6.5%)	46 (8.0%)	20 (5.1%)	2 (3.4%)	0 (0%)	0.11
Daily drinker^‡^	153 (14.6%)	96 (16.7%)	41 (10.5%)	12 (20.3%)	4 (16.7%)	0.03
Low physical activity^‡^	152 (14.5%)	74 (12.8%)	58 (14.8%)	16 (27.1%)	4 (16.7%)	0.03
Low chewing ability^‡^	282 (26.8%)	131 (22.7%)	108 (27.6%)	30 (50.8%)	13 (54.2%)	<0.01
Low swallowing ability^‡^	250 (23.8%)	118 (20.5%)	99 (25.3%)	23 (39.0%)	10 (41.7%)	<0.01
Poor appetite^‡^	365 (34.7%)	167 (29.0%)	171 (43.6%)	16 (27.1%)	11 (45.8%)	<0.01
Social isolation^‡^	433 (41.2%)	250 (43.4%)	145 (37.0%)	28 (47.5%)	10 (41.7%)	0.17
JST-IC*	11 (8–12)	11 (9–13)^a^	10 (8–12)^b^	8 (7–11)^cd^	8 (6–10)^d^	<0.01
Depressive symptoms^‡^	340 (32.4%)	148 (25.7%)	157 (40.1%)	23 (39.0%)	12 (50.0%)	<0.01
Number of comorbidities*	0 (0–1)	0 (0–1)	0 (0–1)	0 (0–1)	1 (0–1)	0.26
Heart disease^‡^	200 (19.2%)	112 (19.6%)	71 (18.3%)	12 (20.3%)	5 (20.8%)	0.95
Stroke^‡^	91 (8.7%)	53 (9.2%)	31 (7.9%)	6 (10.2%)	1 (4.3%)	0.76
Diabetes^‡^	144 (13.7%)	84 (14.6%)	43 (11.0%)	13 (22.0%)	4 (16.7%)	0.09
Cancer^‡^	181 (17.2%)	107 (18.6%)	62 (15.8%)	7 (11.9%)	5 (20.8%)	0.44
Hospitalizations within the prior 12 months^‡^	138 (13.1%)	81 (14.1%)	46 (11.7%)	9 (15.3%)	2 (8.3%)	0.61
LTCI certification^‡^	72 (6.9%)	20 (3.5%)	42 (10.7%)	6 (10.2%)	4 (16.7%)	<0.01

BMI = body mass index, CNAQ = Council on Nutrition Appetite Questionnaire, ECW = extracellular water, FFMI = fat-free mass index, FMI = fat mass index, GDS-15 = Geriatric Depression Scale-15, JST-IC = Japan Science and Technology Agency Index of Competence, LTCI = long-term care insurance, MMSE-J = Mini Mental State Examination-Japanese, TBW = total body water, wt = body weight.

*presented as the median (IQR).

^†^presented as the mean (SD).

^‡^presented as *n* (%).

Underlined text indicates data with significant adjusted standardized residuals.

Different superscript letters

^a^, ^b^, ^c^, and ^d^ indicate statistically significant differences between groups.

Table 3 shows the results of the logistic regression analyses for the associations of living arrangements and cognitive status with nutritional status and body composition. In the univariable model, cognitively impaired older persons living alone [living alone (+)/cognitive impairment (+) group] were more likely to have a low serum albumin level [crude OR = 3.00, 95% confidence interval (CI) = 1.32 to 6.83] and low FFMI (crude OR = 3.24, 95% CI = 1.34 to 7.83). Adjustment for potential confounders did not significantly attenuate this association (adjusted OR = 3.10, 95% CI = 1.31 to 7.33 for low serum albumin and adjusted OR = 2.79, 95% CI = 1.10 to 7.06 for low FFMI). Furthermore, univariable and multivariable logistic regression analyses revealed that cognitively impaired older persons living alone had higher ORs of copresence of a low serum albumin level and low FFMI (crude OR = 8.39, 95% CI = 3.03 to 23.22; adjusted OR = 5.53, 95% CI = 1.81 to 16.90).

**Table 3:** The associations of living arrangements and cognitive status with nutritional status and body composition.

Statistical model: Logistic regression model										
			Univariable model				Multivariable adjusted model*			
Outcome:	Exposure:		Crude OR	95% CI		*p*-value	Adjusted OR	95% CI		*p*-value
Low serum albumin level	Living arrangements and cognitive status									
(serum albumin <4 g/dl)	Living alone (-)	Cognitive impairment (-)	Ref.				Ref.			
	Living alone (+)	Cognitive impairment (-)	1.32	(0.99 to	1.76)	0.05	1.30	(0.94 to	1.80)	0.11
	Living alone (-)	Cognitive impairment (+)	1.78	(1.02 to	3.12)	0.04	1.58	(0.87 to	2.84)	0.13
	Living alone (+)	Cognitive impairment (+)	3.00	(1.32 to	6.83)	0.01	3.10	(1.31 to	7.33)	0.01
Outcome:	Exposure:		Crude OR	95% CI		*p*-value	Adjusted OR	95% CI		*p*-value
Low FFMI	Living arrangements and cognitive status									
(FFMI <16 kg/m^2^ in men and <14 kg/m^2^ in women)	Living alone (-)	Cognitive impairment (-)	Ref.				Ref.			
	Living alone (+)	Cognitive impairment (-)	1.34	(0.94 to	1.91)	0.11	1.25	(0.83 to	1.88)	0.28
	Living alone (-)	Cognitive impairment (+)	2.41	(1.29 to	4.49)	0.01	1.97	(1.02 to	3.82)	0.04
	Living alone (+)	Cognitive impairment (+)	3.24	(1.34 to	7.83)	0.01	2.79	(1.10 to	7.06)	0.03
Outcome:	Exposure:		Crude OR	95% CI		*p*-value	Adjusted OR	95% CI		*p*-value
Low serum albumin and low FFMI	Living arrangements and cognitive status									
	Living alone (-)	Cognitive impairment (-)	Ref.				Ref.			
	Living alone (+)	Cognitive impairment (-)	1.72	(0.95 to	3.09)	0.07	1.58	(0.80 to	3.09)	0.19
	Living alone (-)	Cognitive impairment (+)	3.95	(1.67 to	9.32)	<0.01	1.94	(0.73 to	5.12)	0.18
	Living alone (+)	Cognitive impairment (+)	8.39	(3.03 to	23.22)	<0.01	5.53	(1.81 to	16.90)	<0.01

CI = confidence interval, FFMI = fat-free mass index, OR = odds ratio.

*Adjusted for age, sex, years of schooling, annual income, smoking status, alcohol consumption, physical activity level, chewing ability, swallowing ability, appetite, social isolation, instrumental activities of daily living, depressive symptoms, number of comorbidities, hospitalizations within the prior 12 months, and long-term care insurance certification.

On the other hand, there were no statistically significant interactions between living arrangements and cognitive status in any regression model (S1 Table).

## Discussion

In this cross-sectional study involving community-dwelling men and women aged ≥70 years, we investigated nutritional status and body composition in relation to living arrangements and cognitive status. Compared to the study participants who lived with someone and had normal cognitive function, cognitively impaired persons living alone were approximately 3.1 times more likely to have a low serum albumin level and 2.8 times more likely to have a low FFMI. Both low serum albumin and low fat-free mass levels have been reported as markers of disability and death among older adults [2, 24, 35]. The number of community-dwelling older adults who both live alone and have cognitive impairment will increase with population aging [7–9, 17], and these individuals may be at increased risk of future adverse events.

In our investigation, when limited to populations with cognitive impairment (n = 83), the percentage of older adults living alone was 28.9% (24 out of 83 individuals with cognitive impairment were living alone). Based on reports in the United States, United Kingdom, and Germany, approximately 15 to 50% of community-dwelling cognitively impaired older adults live alone [36–38]. Although direct comparison is difficult due to differences in social and cultural backgrounds, health insurance systems, survey periods, and definitions of cognitive dysfunction, the figure in our study is in the range of those from other countries.

In a cross-sectional study with 6,528 adults (mean age: 72.4 years), the mean (SD) serum albumin level was 4.2 (0.3) g/dL [39], which was comparable to that in our study. A pooled analysis of a cohort of 4478 community-dwelling older Japanese adults (mean age: 72.9 years) [26] reported that the mean (SD) FFMI was 17.4 (1.5) kg/m^2^ in men and 15.3 (1.2) kg/m^2^ in women, and these values were comparable to those in our study (17.3 [1.5] kg/m^2^ in men and 15.2 [1.3] kg/m^2^ in women).

The findings of this study agree with those of previous studies. Cross-sectional studies of older adults have revealed that living alone was associated with poor nutritional status [15, 40]. Other studies demonstrated that cognitively impaired older adults were likely to have poorer nutritional status [11, 13]. Unlike previous studies investigating the individual associations of living alone or cognitive impairment with malnutrition, we investigated the combined effect of living alone and having cognitive impairment, which was a novel aspect of this study. As a result, we found that older adults who both lived alone and had cognitive impairment had a high prevalence of low serum albumin and low FFMIs. The effect of a single factor (i.e., either living alone or having cognitive impairment) was smaller than their combined effect. Nonetheless, we did not find synergistic interactions between living alone and having cognitive impairment. Overall, these results suggest that the combined effect of living alone and having cognitive impairment was additive.

We can assume the reasons for the current study findings that cognitively impaired older adults living alone had poorer nutritional status as follows. Living alone can lead to eating alone and a lack of enjoyment in eating and a lack of appetite [15, 41–43], eventually having adverse effects on nutritional status through disturbances to a balanced diet and the regularity of meals (e.g., skipping meals) [43]. Living alone also has negative effects on psychological and mental health aspects [43], which has been shown to be associated with malnutrition [44, 45]. Cognitive impairment can lead to altered appetite, deterioration of portion control and cooking skills and disturbances to a balanced diet and to the regularity of meals [12, 46–48], which ultimately leads to unfavorable effects on nutritional status. Even if older persons live alone, if their cognitive function is maintained, they can take preventive action to eat healthily and to interact with others on their own. On the other hand, even if the cognitive status of older persons has declined, if they live with someone, they can receive support or encouragement from others whom they are living with regarding the preparation of meals that prevent malnutrition. However, if the cognitive status of older persons declines and if they live alone, they are less likely to have the opportunity to obtain such support. Consequently, cognitively impaired older adults living alone are at increased risk of malnutrition.

Notably, the associations of living arrangements and cognitive status with nutritional status and body composition were not greatly attenuated after adjustment for the social isolation variable in our analyses. In our study, study participants who answered “no” to the question “Do you have contact at least once a week with anyone, including relatives living apart, friends, and neighbors?” were categorized as socially isolated (Table 1) [19]. There is a possibility that this categorization did not reflect social support in meal preparation and/or cooking. Therefore, this variable did not mediate the effects of living alone and cognitive impairment on nutritional status in our study. In addition, although we considered the status of LTCI certification in the analyses, we did not obtain data on meal preparation or delivery service based on LTCI; therefore, we did not consider formal care services regarding their meals. Further studies with data on social support from nutritional aspects are needed.

Living arrangements and cognitive status were associated with chewing and swallowing abilities and depressive symptoms. These findings suggest that oral health or mental health intervention may be effective strategies for preventing malnutrition, and these effects should be investigated in future studies.

A serum albumin level of 3.5 g/dL was the common lower limit threshold. We found that only 0.5% of the study population (n = 5) had a serum albumin level of <3.5g/dL. Previous studies have found that a cut-off point of 4.0 g/dL predicts the risks of disability and mortality in community-dwelling older adults [25, 49]. Therefore, we used a cut-off point of serum albumin level of 4.0 g/dL.

The present study has several strengths. The first distinguishing feature of this study is its rich data on demographic, socioeconomic, and medical characteristics, collected from over a thousand community-dwelling adults. Second, trained nurses and clinical psychologists carried out the cognitive assessments, increasing the validity of exposure. Third, the outcomes for nutritional status and body composition were objectively assessed and were thus not affected by memory bias.

We revealed novel findings that cognitively impaired persons living alone had low serum albumin levels. In Japan, serum albumin levels are used to assess malnutrition risks among individuals aged ≥65 years old [50]. However, notably, nutritional status is not precisely assessed through serum albumin levels alone. Serum albumin is an indicator of nutritional state, but it is very unspecific [51]. There are etiology-based types of malnutrition; disease-related malnutrition with and without inflammation; and malnutrition/undernutrition without disease [1]. We could not classify the state of the malnutrition based on the available data. To explore the association of living arrangements and cognitive status with nutritional status classification stated above is an important next step. In addition, the present study had other limitations that must be considered. First, the study population consisted of a convenient sample who voluntarily participated in the questionnaire survey and health examinations conducted at a healthcare facility. We therefore may have underestimated the prevalence of cognitively impaired older adults living alone in our survey area and underestimated the effects of living alone and cognitive impairment on nutritional status. Although we found statistically significant results, the small number of participants who both lived alone and had cognitive impairment prevented us from performing further analysis, such as stratification analysis. In addition, the study population included only Japanese older adults from one specific area. Therefore, the results may not be generalizable to other populations. Future studies with larger sample sizes and broader populations are required. Second, since the study had a cross-sectional design, the temporal associations of living alone and cognitive impairment with nutritional status could not be assessed. Notably, some of the associations in our analyses might have had the opposite effect from that hypothesized. For example, cognitive impairment is a potential consequence of poor nutrition [52]. Thus, longitudinal analyses must be conducted when the follow-up dataset is available. Finally, as with any multivariable analyses, residual confounding due to unmeasured variables, such as the presence of formal/informal caregivers and social service utilization patterns and/or unexpected confounding, might have existed.

In conclusion, cognitively impaired older adults living alone had poorer nutrition than older adults who lived with someone and had normal cognitive function. Our results highlight the importance of paying extra attention to nutritional status for this group of community-dwelling older adults. However, further studies must be conducted to validate the results of the current study.

## Supporting information

S1 Table Logistic regression analyses of the interactions between living arrangements and cognitive status on nutritional status and body composition (n = 1051). (DOCX)
