# Supplementary material for: Nutritional status and body composition in cognitively impaired older persons living alone: The Takashimadaira study
Source: PLoS One. 2021 Nov 23;16(11):e0260412. doi: 10.1371/journal.pone.0260412 (PMC8610283; doi:10.1371/journal.pone.0260412)
Supplement: S1 Table — (DOCX) [file pone.0260412.s001.docx]

Supporting information captions

**S1 Table. Logistic Regression Analyses of the Interactions between Living Arrangements and Cognitive Status on Nutritional Status and Body Composition (n=1051).**

**S1 Table. Logistic Regression Analyses of the Interactions between Living Arrangements and Cognitive Status on Nutritional Status and Body Composition (n=1051).**

| Statistical model: Logistic regression model | | | | | | | | | | |
| --- | --- | --- | --- | --- | --- | --- | --- | --- | --- | --- |
|  |  | Model 1 (crude model) | | | |  | Model 2 (multivariable adjusted model)^*^ | | | |
| Outcome | Exposure | OR | 95%CI | | *p*-value |  | OR | 95%CI | | *p*-value |
| Low serum albumin level | Living arrangements and cognitive status | |  |  |  |  |  |  |  |  |
| (serum albumin <4g/dl) | Living alone | 1.32 | (0.99 to | 1.76) | 0.05 |  | 1.30 | (0.94 to | 1.80) | 0.11 |
|  | Cognitive impairment | 1.78 | (1.02 to | 3.12) | 0.04 |  | 1.58 | (0.87 to | 2.84) | 0.13 |
|  | Living alone × Cognitive impairment | 1.27 | (0.47 to | 3.45) | 0.64 |  | 1.51 | (0.54 to | 4.21) | 0.43 |
|  | | | | | | | | | | |
| Outcome | Exposure | OR | 95%CI | | *p*-value |  | OR | 95%CI | | *p*-value |
| Low FFMI | Living arrangements and cognitive status | |  |  |  |  |  |  |  |  |
| (FFMI <16 kg/m^2^ in men and <14 kg/m^2^ in women) | Living alone | 1.34 | (0.94 to | 1.91) | 0.11 |  | 1.25 | (0.83 to | 1.88) | 0.28 |
|  | Cognitive impairment | 2.41 | (1.29 to | 4.49) | 0.01 |  | 1.97 | (1.02 to | 3.82) | 0.04 |
|  | Living alone × Cognitive impairment | 1.01 | (0.34 to | 2.98) | 0.99 |  | 1.13 | (0.37 to | 3.48) | 0.83 |
|  | | | | | | | | | | |
| Outcome | Exposure | OR | 95%CI | | *p*-value |  | OR | 95%CI | | *p*-value |
| Low serum albumin level and low FFMI | Living arrangements and cognitive status | |  |  |  |  |  |  |  |  |
|  | Living alone | 1.72 | (0.95 to | 3.09) | 0.07 |  | 1.58 | (0.80 to | 3.09) | 0.19 |
|  | Cognitive impairment | 3.95 | (1.67 to | 9.32) | 0.00 |  | 1.94 | (0.73 to | 5.12) | 0.18 |
|  | Living alone × Cognitive impairment | 1.24 | (0.33 to | 4.66) | 0.75 |  | 1.81 | (0.43 to | 7.53) | 0.41 |

CI = confidence interval, FFMI = fat-free mass index, OR = odds ratio

^*^Adjusted for age, sex, years of schooling, annual income, smoking status, alcohol consumption, physical activity level, chewing ability, swallowing ability, appetite, social isolation, instrumental activities of daily living, depressive symptoms, number of comorbidities, hospitalizations within the prior 12 months, and long-term care insurance certification.
